# Diagnostic and prognostic impact of serum HE4 detection in endometrial carcinoma patients

**DOI:** 10.1038/bjc.2011.109

**Published:** 2011-04-05

**Authors:** E Bignotti, M Ragnoli, L Zanotti, S Calza, M Falchetti, S Lonardi, S Bergamelli, E Bandiera, R A Tassi, C Romani, P Todeschini, F E Odicino, F Facchetti, S Pecorelli, A Ravaggi

**Affiliations:** 1 ‘Angelo Nocivelli’ Institute of Molecular Medicine, Department of Obstetrics and Gynecology, Division of Gynecologic Oncology, University of Brescia, Viale Europa 11, 25123, Brescia, Italy; 2 Section of Medical Statistics and Biometry, Department of Biomedical Sciences and Biotechnology, University of Brescia, Viale Europa 11, 25123, Brescia, Italy; 3 Department of Pathology, University of Brescia, Viale Europa 11, 25123, Brescia, Italy

**Keywords:** serum marker, endometrial carcinoma, HE4, diagnosis, prognosis

## Abstract

**Background:**

To date, no good marker for screening or disease monitoring of endometrial cancer (EC) is available. The aims of this study were to investigate HE4 gene, protein expression and serum HE4 (sHE4) levels in a panel of ECs and normal endometria (NEs) and to correlate sHE4 with patient clinicopathological characteristics and prognosis.

**Methods:**

Using quantitative real-time PCR we tested 46 ECs and 20 NEs for *HE4* gene expression. Protein expression was analysed by immunohistochemistry on tissue microarrays in 153 ECs and 33 NEs. Pre-operative serum samples from 138 EC and 76 NE patients were analysed with HE4–EIA assay. Association between sHE4 and patient clinicopathological characteristics or outcome was evaluated.

**Results:**

Protein and HE4 gene were significantly upregulated in EC tissues and sera, compared with controls. High sHE4 levels were significantly associated with worse EC clinical characteristics. By univariate survival analysis, high sHE4 levels significantly correlated with decreased overall survival, progression-free survival and disease-free survival, retaining their independent prognostic value on the poorly differentiated EC cohort.

**Conclusion:**

We demonstrate, for the first time, that high sHE4 levels correlates with an aggressive EC phenotype and may constitute an independent prognostic factor for poorly differentiated-ECs. Determination of sHE4 could be clinically useful in identifying high-risk EC patients for a more aggressive adjuvant therapy.

Endometrial carcinoma (EC) is the most common gynaecological malignancy in the Western world. (Sherman, 2000; Amant *et al*, 2005). Most EC patients have oestrogen-related tumours, well-differentiated, endometrioid in histology and, consequently, with good prognosis (Type I ECs). In contrast, Type II ECs include non-oestrogen-related poorly differentiated endometrioid, serous papillary and clear-cells ECs, which being aggressive and metastatic at presentation, often recur despite aggressive clinical interventions (Bokhman, 1983). Unfortunately, to date, no good marker for EC screening, early diagnosis or disease monitoring is available. In this regard, CA125 serum determination is often used in clinical practice to monitor EC patients (Duk *et al*, 1986). However, this marker appears to have limited utility in analysing the effects of adjuvant therapy or in the prediction of tumour recurrence. Endometrial carcinoma conventional prognostic factors are tumour grade, International Federation of Gynecology and Obstetrics (FIGO) staging and histological type (Bokhman, 1983; Cirisano *et al*, 1999, 2000; Creasman *et al*, 2001; Gemer *et al*, 2004), but they are insufficient to identify patients with poor prognosis. The recent progress in molecular biology has identified novel biological markers (Kohler *et al*, 1996; Risinger *et al*, 1998; Maxwell *et al*, 2001; Morrison *et al*, 2006) representing important alternative indicators of patients with biologically aggressive, high-risk tumours, which may benefit from adjuvant therapies to improve outcome (Greven *et al*, 2006; Randall *et al*, 2006).

Human epididymis protein HE4 (WFDC2) was first identified by Kirchhoff *et al* (1991) and specifically localised to the epithelial cells of the epididymal duct. More recently, HE4 expression has been reported in a number of normal human tissues outside of the male reproductive system, as well as in various types of human carcinomas, including EC (Bingle *et al*, 2002; Galgano *et al*, 2006). A recent paper of Moore *et al* (2008) demonstrated that HE4 preoperative serum levels discriminate EC patients from healthy postmenopausal women, suggesting its promising value as diagnostic serological marker to be used alone or in combination.

In the present report, we have analysed HE4 gene expression levels by quantitative real-time PCR, whereas protein expression was tested by immunohistochemistry on tissue microarrays in a large cohort of EC patients. Furthermore, with the aim to confirm HE4 potential utility in EC diagnosis, using a commercially available ELISA assay, we have measured preoperative HE4 serum levels in a large cohort of EC patients and in healthy controls. In addition, we have compared HE4 serum levels with those of CA125, the marker more often used in clinical practice. Finally, we have investigated the correlation between HE4 serum levels and either clinical factors or survival end points to determine its potential prognostic significance. The univariate and multivariate survival analyses were performed both on the entire cohort of EC patients and on the poorly differentiated EC subgroup.

## MATERIALS AND METHODS

### Human tissue and serum samples and patient characteristics

EC tissue samples were obtained from 153 patients treated at the Division of Gynecologic Oncology of the University of Brescia, Italy between September 2003 and July 2009. Moreover, samples of normal endometria (NE) were collected from 33 age-matched patients, undergoing surgery for benign pathologies. Preoperative serum samples from 138 patients with EC and from 76 controls (postmenopausal women without gynaecological pathologies) were collected. The study was approved by the Institutional Review Board and written informed consent was obtained from all the patients enrolled. All pathology specimens were reviewed in our institution and histological classification was performed according to the WHO criteria, whereas pathological stage was determined in accordance with the FIGO guidelines. Tumour tissues were obtained from women undergoing total abdominal hysterectomy, bilateral salpingo-oophorectomy and peritoneal washings for cytology. Lymph node sampling or dissection was predominantly performed in patients with tumours characterised by deep myometrial invasion and/or high-grade or aggressive histology. The presence of serious concomitant diseases, obesity and advanced age were contraindications to full-surgical staging. None of the patients had received preoperative chemotherapy or radiation. Age, histological type, stage, grade, additional disease (i.e., diabetes, obesity and blood pressure) and treatment information were recorded in all cases. A group of 134 patients was selected for survival analysis. Patients were followed up from the date of surgery until death or the last observation (median follow-up, 33.1 months, range 0.9–74.4 months). At the time of the last follow-up, 107 patients (79.9%) were alive without evidence of disease, 8 patients (6.0%) were alive with disease, 18 patients (13.4%) were dead from disease and 1 died from other causes. For subgroup survival analysis, 54 patients harbouring poorly differentiated ECs were considered.

### Total RNA extraction and quantitative real-time PCR

Tissue-sharp dissection, liquid nitrogen freezing and epithelial purity checking of EC and NE samples were performed as previously reported (Bignotti *et al*, 2006), as well as total RNA extraction and reverse transcription. Quantitative real-time PCR was performed in triplicate as previously reported (Bignotti *et al*, 2006), using the following Assay on Demand (Applied Biosystems, Foster City, CA, USA): Hs00899484_m1 (WFDC2). Normal and EC samples were analysed at the same time to avoid batch effects. Data were normalised using glyceraldehyde-3-phosphate dehydrogenase as internal control.

### Tissue microarrays and immunohistochemistry

Tissue microarray blocks (TMAs) were created from 153 formalin-fixed, paraffin-embedded EC and 33 NE tissues collected from the Department of Surgical Pathology of the University of Brescia, Italy. Tissue microarray blocks were constructed using an automated tissue microarrayer (TMA Master, 3DHistech, Budapest, Hungary). Representative areas were chosen for sampling from haematoxylin and eosin (H&E) stained sections of selected NE and EC cases of different histological subtypes. Four 0.6-mm cores have been collected from different areas of each tumour block in order to overcome tumour heterogeneity and the possible loss of tissue because of cutting. Different TMAs were created for low and advanced tumour grade, and normal tissue was included in all TMAs to be used as control. Sections (4 *μ*m thick) were cut from TMAs and H&E staining were used for confirmation of tumour tissue. Tissue microarray sections were subjected to antigen retrieval (15 min in microwave oven at 750 W in sodium citrate buffer, pH 7.0), before application of the rabbit polyclonal antibody to HE4 (1 : 40 dilution; Covance, Dedham, MA, USA). The antibody was revealed with NovoLink polymer (Novocastra Laboratories, Newcastle upon Tyne, UK), followed by diaminobenzidine as chromogen; hematoxylin was used for counterstaining. Immunoreactivity was evaluated by four independent observers; cytoplasmic staining was graded for intensity (0-negative, 1-weak, 2-moderate and 3-strong) and the percentage of positive cells was scored as 0 (0%), 1 (1–10%), 2 (11–50%) and 3 (51–100%). A single scale with scores 0–9 was obtained by multiplying the intensity and the percentage staining score, and a total score was calculated by grouping score 0 in total score 0, 1–3 in total score 1, 4 and 6 in total score 2 and 9 in total score 3. Digital images were resized by using Adobe Photoshop (Element version) in order to have homogeneous cores (about 90% of the full core is represented).

### HE4 Immunoassay

All serum samples were collected, before any patient treatment, frozen in liquid nitrogen within 2 h of blood drawing and stored at −80°C. Serum HE4 levels were measured using HE4 EIA kit (Fujirebio Diagnostics, Inc., Goteborg, Sweden), following manufacturer's instruction. Normal and EC samples were analysed in duplicate at the same time to avoid batch effects.

### CA125 serum level measurements

Serum CA125 values were determined by the clinical laboratory at the Spedali Civili di Brescia, Italy using the Architect CA125 II chemiluminescent two step immunoassay kit (Abbott Diagnostics, Abbott Park, IL, USA), following the manufacturer's protocol. Normal and EC samples were analysed at the same time to avoid batch effects.

### Statistical analysis

In all the analyses, HE4 gene expression has been considered on log-scale. Differences in HE4 gene expression between ECs and NEs were evaluated with *t*-test, whereas IHC values were tested with the Wilcoxon rank sum test and Mann–Whitney *U*-test. Differences in HE4 serum levels between the groups were calculated using ANOVA. Spearman's rank correlation was used to estimate the degree of association between serum HE4 (sHE4) and CA125 values, whereas Kendall's coefficient was used to estimate the degree of concordance between IHC and ELISA data. Combination of markers was investigated comparing ROC curves, based on the method proposed by DeLong *et al* (1988). Different ROCs are built, based on predicted values for each sample derived from logistic models, accounting for different combinations of markers. Predicted values are computed using leave-one-out cross validation. The association between HE4 serum levels and clinicopathological parameters was investigated with ANOVA. For survival analysis, three end points (cancer relapse, cancer progression and death due to cancer) were used to calculate disease-free survival (DFS), progression-free survival (PFS) and overall survival (os), respectively. Disease-free survival was defined as the time interval between the date of surgery and the date of identification of disease recurrence, PFS was defined as the time interval between the date of surgery and the date of identification of progressive disease (disease not treatable with curative intent) and OS was defined as the time interval between the date of surgery and the date of death. For all three end points, the last date of follow-up was used for censored subjects. Survival models were fitted using the Cox proportional hazard models, whereas survival curves were drawn based on the Kaplan–Meier methods. The effect of HE4 serum levels on prognosis was evaluated by categorising the values in tertiles computed on the entire cohort (low; medium; high). In all the analyses, a *P*-value<0.05 was considered significant. All the analyses were performed using *R* (R Development Core Team, 2010).

## RESULTS

### HE4 gene expression by quantitative RT–PCR

**Figure 1:**
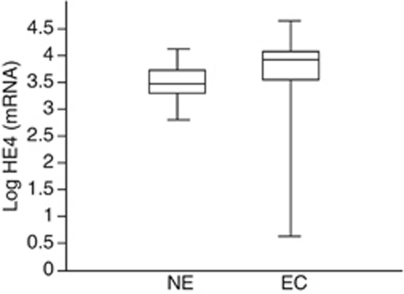
HE4 mRNA expression in endometrial carcinoma (EC) compared with normal endometrial tissues (NE). The figure shows the box plot of the relative quantification values in Log scale. As shown, HE4 mRNA expression was significantly higher in EC compared with NE patients (*P*<0.0001).

Human epididymis protein HE4 gene expression was tested by qRT–PCR in 46 ECs (20 poorly differentiated (G3), 17 moderately differentiated (G2) and 9 well differentiated (G1)) and 20 NEs. As shown in Figure 1, HE4 mRNA expression was significantly higher in EC compared with NE patients (FC=2.63, 95% CI: 1.78–3.88, *P*<0.0001).

### HE4 protein expression by immunohistochemical staining

**Figure 2:**
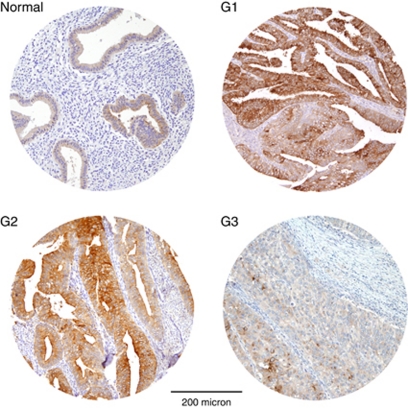
Representative immunohistochemical staining for HE4 in tissue microarrays of normal endometria (normal) and endometrial carcinomas (G1, G2 and G3). Normal tissues show predominantly a weak immunoreactivity for HE4 (mostly 1+), whereas G1, G2 and G3 endometrial carcinomas are mainly scored 3+, 2+ and 0/1+, respectively. Magnification: × 100; scale bar length: 200 micron.

To analyse HE4 expression results at the protein level, immunohistochemistry for HE4 was carried out on 153 EC (53 G3, 60 G2 and 40 G1, including 119 endometrioid ECs and 34 non-endometrioid ECs) and 33 NE (16 during proliferative phase and 17 during secretory phase) TMAs. As shown in Table 1, a positive staining for HE4 was detected in 130 out of 153 (85.0%) ECs and appeared to be moderate/strong (score 2/3) in 59.5% of cases. Normal endometria tissues predominantly showed a weak immunoreactivity, with presence of score 1 in 57.6% of cases. Endometrial cancers showed markedly increased HE4 positivity as compared with NEs (*P*=0.03). As represented in Figure 2, HE4 staining in EC and NE samples appeared to be cytoplasmic and restricted to the epithelial compartment, with no positivity in adjacent stromal cells.

As displayed in Table 1, all G1 cases were positive and most of them (31 out of 40, 77.5%) showed a cytoplasmic staining with moderate/strong score. Most of G2 cases (53 out of 60, 88.3%) were also found positive for HE4 and showed a moderate/strong score in 65% of cases, whereas G3 tumours (32 out of 53, 60.4%) were mainly scored negative/weak. Immunostaining of HE4 was significantly greater in G1 and in G2 tumours compared with G3 ones (*P*<0.0001 and *P*=0.0062, respectively). Moreover, immunoreactivity for HE4 was significantly stronger in endometrioid ECs compared with non-endometrioid ECs (*P*=0.016).

### Serum HE4 levels

**Figure 3:**
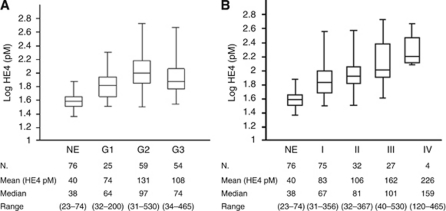
Box plots showing serum HE4 (sHE4) levels in controls with normal endometrium (NE) and in endometrial cancer patients, represented according to differentiation grade (**A**) and to FIGO stage (**B**).

Serum samples were collected from 138 EC patients and 76 healthy controls and tested with HE4 ELISA. Median, mean and range values for each group are displayed in Figures 3A and B. As shown, sHE4 levels were significantly higher in EC patients compared with NEs (median ECs=83 pM, median NEs=38 pM, FC=2.33, *P*<0.0001, 95% CI: 2.02–2.73), regardless of the FIGO stage (NEs *vs* Stage I ECs, *P*=0.004; NEs *vs* stage II, III or IV ECs, *P*<0.0001) and differentiation grade (NEs *vs* G1, G2 or G3 ECs, *P*<0.0001). Moreover, sHE4 levels in G1 ECs showed a significant difference with G2 ECs (FC=1.64, *P*=0.0009, 95% CI: 1.22–2.20). The comparisons between G2 ECs and G3 ECs and between G1 ECs and G3 ECs were not significant (*P*=0.240 and *P*=0.066, respectively). In addition, sHE4 levels were higher in patients with advanced FIGO stages: I–II (mean 90) *vs* III–IV stages (mean 174), *P*=0.014. Finally, sHE4 levels and IHC results showed a low concordance in paired tumour samples (*P*=0.18, *W*=0.563).

### Comparison between HE4 and CA125 serum levels and combination of the two markers

We analysed CA125 serum levels in 127 EC patients and in 71 NEs, all tested with HE4 ELISA. Analysed CA125 median values were 11 U (range 4–42), 12 U (range 3–40), 20 U (range 1–202) and 19 U (range 4–853) for NEs, G1, G2 and G3 ECs, respectively. Median values of CA125 were 15 U (range 1–114), 15 U (range 4–50), 39 U (range 4–202) and 67 U (range 18–853) for stage I, stage II, stage III and stage IV ECs, respectively. The difference between serum CA125 levels in NEs compared with all ECs was statistically significant (median ECs=18 U, median NEs=11 U, *P*<0.0001), whereas it was not significant when comparing NEs with G1 ECs (*P*=0.99), NEs with stage I ECs (*P*=0.95) and NEs with stage II ECs (*P*=0.93). Logistic models were used to compare the sensitivity of CA125 and HE4 markers for the differentiation of ECs *vs* NEs at set specificities of approximately 90, 95 and 98% (Table 2). HE4 showed a considerably higher sensitivity compared with CA125 for detecting EC, considering all stages and all set specificities. For instance, HE4 had a sensitivity of 67% at a specificity of 95% compared with 30% for CA125. For stage I cancers, HE4 exhibited a 39% improvement in sensitivity at a specificity of 95% compared with CA125 alone (Table 2). As CA125 and HE4 serum levels were significantly correlated, but exhibited a low Spearman's rank coefficient (*P*<0.01, rs=0.38), a combination of the two markers was analysed. The combination of CA125 and HE4 led to a further improvement in sensitivity, even if limited, compared with HE4 alone, considering all set specificities and all EC stages. Examining only stage I EC patients, there was no gain in sensitivity when CA125 and HE4 were combined compared with HE4 alone.

### sHE4 levels and clinicopathological variables

The relationship between sHE4 levels and the clinicopathological features of the 138 EC patients is shown in Table 3. Higher sHE4 levels were significantly associated with advanced age at diagnosis, menopause, higher FIGO stage and grade, deeper myometrial invasion, positive lymph nodes, presence of lymphovascular invasion, cervical and adnexal involvement, positive peritoneal cytology and administration of either chemotherapy or adjuvant radiation therapy.

### sHE4 levels and patient survival

**Figure 4:**
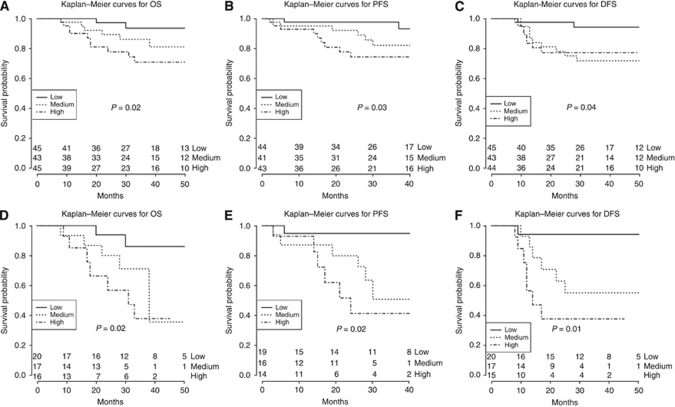
Kaplan–Meier survival curves for EC patients according to sHE4 levels on the entire patient cohort (**A**, overall survival; **B**, progression-free survival; **C**, disease-free survival) and on poorly differentiated subgroup of EC patients (**D**, overall survival; **E**, progression-free survival; **F**, disease-free survival). *P*-values refer to the comparison between high *vs* low tertile, except for **C**, in which medium *vs* low tertile shows to be significant.

As expected, known EC clinical prognostic factors such as FIGO stage, histological type and lymph node involvement showed a statistically significant association with OS, PFS and DFS in univariate analyses (all *P*<0.05, data not shown), proving the validity of the patient cohort recruited in this study. In addition, as displayed in Figures 4A and B, respectively, higher HE4 serum levels (high *vs* low HE4 tertiles) showed a significant association with poor OS (*P*=0.02) and shorter PFS (*P*=0.03). Regarding DFS (Figure 4C), medium *vs* low HE4 tertiles was significantly correlated with decreased DFS (*P*=0.04), whereas the difference between high and low HE4 tertiles showed a marginal significance (*P*=0.06). FIGO stage, histological type, lymph node involvement and HE4 serum levels were then included in a multivariate analysis. Non-endometrioid EC histological subtype, along with advanced FIGO stage, were identified as independent predictive factors for poor OS (*P*=0.01 and *P*=0.04, respectively, Table 4A), whereas sHE4 levels (medium *vs* low tertile) were shown to be marginally significant as prognostic factor for shorter OS (*P*=0.08, Table 4A). Regarding PFS, only histological type and, marginally, FIGO stages were of prognostic significance, whereas sHE4 levels were not (Table 4A). Regarding DFS, neither clinical parameters nor sHE4 levels were indicative of disease recurrence, even if elevated sHE4 levels exhibited the highest trend toward significance (*P*=0.14 and *P*=0.17, Table 4A). Then we performed a further survival analysis in the subgroup of 54 patients harbouring poorly differentiated ECs. The univariate model revealed that patients with elevated sHE4 levels (high tertile) had a significant poorer OS (*P*=0.02, Figure 4D), shorter PFS (*P*=0.02, Figure 4E) and worse DFS (*P*=0.01, Figure 4F) than patients with reduced sHE4 levels (low tertile). In multivariate analysis, sHE4 levels retained its significance as an independent prognostic factor for poor OS (*P*=0.04), shorter PFS (*P*=0.04) and decreased DFS (*P*=0.01) in the subgroup of patients with poorly differentiated ECs (Table 4B).

## DISCUSSION

Endometrial carcinoma (EC) is generally considered a malignancy with favourable prognosis, because of the fact that the majority of patients declare their disease early by postmenopausal bleeding and therefore can be diagnosed at the first stage. However, an accurate serum marker for screening and early diagnosis would certainly be useful for those patients that may experience an increased risk of developing EC, such as those with severe obesity and diabetes, Lynch syndrome, PTEN gene defects or breast cancer women on Tamoxifen. Moreover, several high-risk EC groups, such as women with stages III–IV disease or even stage I patients harbouring high-grade carcinomas, or deep myometrial invasion, would benefit from a marker to give pre-operative indications, to monitor the effects of adjuvant therapy and to predict early tumour recurrence. Serum levels of CA125 are commonly used in the clinic for these purposes, but exhibit a low sensitivity and specificity (Sood *et al*, 1997; Powell *et al*, 2005). In the present investigation, with the aim to fully characterise human epididymis protein HE4 as a marker for EC, we have analysed its gene and protein expression in tumour tissues, its secretion in the sera and, finally, its prognostic value on a cohort of well-characterised EC patients. In a previous microarray study, Huhtinen *et al* (2009) examined HE4 gene expression in a limited number of EC samples, finding no significant difference with NEs. Conversely, according to our gene expression results, HE4 mRNA was significantly upregulated in ECs compared with NEs. Moreover, we demonstrated a significantly stronger HE4 immunohistochemical staining in EC compared with NE tissues. HE4 immunostaining in NE predominantly exhibits a weak score, whereas most EC samples show moderate/strong scores. Those results are in agreement with two previous reports regarding HE4 immunohistochemistry performed on a limited number of EC and NE samples (Drapkin *et al*, 2005; Galgano *et al*, 2006). Few recent studies have proposed HE4, a candidate molecular marker for ovarian cancer, as a promising serum marker for endometrial malignancies (Moore *et al*, 2008; Huhtinen *et al*, 2009; Montagnana *et al*, 2009). Serum CA125 and HE4 median values in EC and NE patients were shown to be similar in our investigation compared with previous studies, thus validating our patient cohort. In agreement with Moore *et al* (2008) we observed significantly higher sHE4 levels in EC patients when compared with healthy women. Considering all EC stages, sHE4 sensitivity resulted to be higher than that of sCA125 in detecting cancer patients and, more importantly, we obtain the same result when sHE4 is tested for the detection of stage I EC. Interestingly, our study showed increased HE4 sensitivities at every specificities compared with those reported in the literature (Moore *et al*, 2008), regardless of the EC stage. Finally, the addition of HE4 to CA125 significantly raised the sensitivity compared with CA125 alone, either in the entire EC cohort (68 *vs* 30% at 95% specificity, respectively), or in stage I ECs (53 *vs* 15% at 95% specificity, respectively). Immunohistochemical staining data and sHE4 levels showed a low concordance coefficient in paired tumour samples. This discrepancy may be partially explained by the IHC semi-quantitative scoring system and by the probable heterogeneity of the tumour tissue. Furthermore, this finding suggests that HE4 ELISA, that is a dynamic test to be performed at any time and offers precise marker quantification in serum, should be preferred to tissue immunostaining in EC clinical setting.

The HE4 higher sensitivity over CA125 in identifying stage I ECs indicates its potential prognostic value in detecting early tumour recurrence. There are limited evidence reporting sHE4 as a predictive marker for recurrence in ovarian cancer (Havrilesky *et al*, 2008; Anastasi *et al*, 2010), whereas to our knowledge, so far no prognostic value of sHE4 levels in EC patients has been investigated. Herein, for the first time we demonstrated the significant association between elevated sHE4 levels and adverse EC factors, which may suggest a relation between increased tumour biological aggressiveness and HE4 release in EC. Moreover, our study is the first to correlate sHE4 levels with clinical outcome in patients with EC. In univariate analysis on the entire EC cohort, we found that higher sHE4 levels were significantly more often observed in patients with poor OS, PFS and DFS. The multivariate analysis showed histological type and staging as independent prognostic factor for OS, whereas sHE4 levels did not reach the statistical significance. We then decided to focus our survival analysis on a high-risk subgroup of EC patients, harbouring poorly differentiated ECs, characterised by highly malignant cancers with poor prognosis (Bokhman, 1983). Remarkably, this is the first investigation reporting higher sHE4 levels as the only independent prognostic factor for shortened PFS and DFS in this selected group of patients. Regarding OS, sHE4 levels and histological types were independent prognostic factors, with the former showing a higher hazard ratio compared with the latter. Given these findings and the poor efficacy of current treatment modalities, it seems that patients with poorly differentiated ECs and high sHE4 levels could be managed more aggressively, using contemporary therapeutic options, than those with low sHE4 levels. However, these data should be confirmed with additional studies on larger patient cohort before routine sHE4 levels evaluation could be applied in the clinical setting.

Summarising, our results confirm that HE4 is an accurate and sensitive serum marker for early detection of EC patients, exhibiting a better diagnostic performance compared with CA125, which is the marker conventionally used in EC management. In addition, we demonstrated for the first time that high sHE4 levels may identify patients harbouring a more aggressive EC phenotype and may be an independent prognostic factor for OS, PFS and DFS in poorly differentiated EC patients. Therefore, the evaluation of sHE4 levels might be useful as an early, simple and highly efficient tool to select high-risk EC patients who could benefit from a tailored surgical (i.e., the extension of the lymph nodal dissection) and adjuvant (i.e., the extension of radiotherapy fields, systemic chemotherapy or both) therapy. Large prospective clinical studies are certainly necessary to support these findings and to assess the potential of HE4 as a new tool for preoperative evaluation and postoperative surveillance of EC patients.

## Figures and Tables

**Table 1:** HE4 immunoreactivity in tissue microarrays of endometrial carcinomas (ECs) and normal endometria (NEs)

**HE4 protein expression**					
	***n***	**Score=0 *n* (%)**	**Score=1 *n* (%)**	**Score=2 *n* (%)**	**Score=3 *n* (%)**
*ECs*	153	23 (15.0)	39 (25.5)	61 (39.9)	30 (19.6)
G1	40	0 (0.0)	9 (22.5)	16 (40.0)	15 (37.5)
G2	60	7 (11.7)	14 (23.3)	27 (45.0)	12 (20.0)
G3	53	16 (30.2)	16 (30.2)	18 (34.0)	3 (5.6)
NEs	33	2 (6.0)	19 (57.6)	12 (36.4)	0 (0.0)

Abbreviations: HE4=human epididymis protein 4; EC*=*endometrial carcinoma; NE=normal endometria.

**Table 2:** Tumour biomarker sensitivities among patients with endometrial cancer

	**Controls *vs* stage I EC: sensitivity (%)**			**Controls *vs* all stages EC: sensitivity (%)**		
**Marker**	**90% specificity (%)**	**95% specificity (%)**	**98% specificity (%)**	**90% specificity (%)**	**95% specificity (%)**	**98% specificity (%)**
HE4	61	54	45	75	67	59
CA125	26	15	11	36	30	26
HE4+CA125	61	53	44	76	68	60

Abbreviations: CA125=cancer antigen 125; EC*=*endometrial carcinoma; HE4=human epididymis protein 4.

**Table 3:** Clinical and pathological characteristics of 138 endometrial cancer patients and their association to HE4 serum levels (pM)

**Variable**	***n***	**HE4 median (IQR)**	***P*-value**
*Age at diagnosis (years)*			
<65	60	59 (51.5)	
⩾65	78	96 (82.0)	<0.01
			
*FIGO stage*			
I+II	106	74 (56.0)	<0.01
III+IV	32	112 (117.7)	
			
*WHO grading*			
Grade 1	25	64 (41.0)	
Grade 2+3	113	89 (76.2)	<0.01
			
*Histology*			
Endometrioid	109	88 (79.0)	
Non-endometrioid	29	70 (52.2)	0.68
			
*Myometrial invasion*			
M0+M1	63	66 (42.5)	<0.01
M2	75	98 (93.5)	
			
*Lymph node status*			
Negative	93	74 (53.0)	
Positive	21	97 (108.0)	0.017
Unknown	24		
			
*Cervical involvement*			
Absent	91	74 (53.0)	
Stromal involvement	26	141 (169.7)	<0.01
Unknown	21		
			
*Adnexal involvement*			
Negative	121	82 (64.5)	
Positive	17	95 (167.0)	0.04
			
*Peritoneal cytology*			
Negative	121	82 (59.0)	
Positive	12	126 (161.7)	<0.01
Unknown	5		
			
*Lymphovascular invasion*			
Absent	60	68 (43.7)	<0.01
Present	73	97 (88.7)	
Unknown	5		
			
*Parity*			
Nulliparity	24	82 (66.0)	0.65
Pluriparity	112	84 (69.0)	
Unknown	2		
			
*Body mass index*			
<25	46	89 (82.5)	
⩾25	80	82 (75.0)	0.84
Unknown	12		
			
*Hypertension*			
Negative	58	80 (53.2)	
Positive	78	88 (87.0)	0.09
Unknown	2		
			
*Diabetes*			
Negative	117	85 (58.2)	0.31
Positive	20	95 (92.0)	
Unknown	1		
			
*Menopause*			
Negative	14	55 (57.2)	
Positive	123	88 (68.2)	0.04
Unknown	1		
			
*Smoking*			
Negative	98	82 (71.0)	
Positive	26	77 (46.5)	0.33
Unknown	14		
			
*Radiotherapy*			
Negative	62	66 (44.0)	0.04
Positive	48	96 (68.0)	
Unknown	28		
			
*Chemotherapy*			
Negative	62	66 (44.0)	
Positive	17	120 (177.0)	0.02
Unknown	59		

Abbreviations: FIGO=International Federation of Gynecology and Obstetrics; HE4=human epididymis protein 4; IQR=interquartile range; WHO=World Health Organisation.

**Table 4:** Multivariate analyses of OS, DFS and PFS in relation to clinical parameters and HE4 serum levels (A, entire cohort of EC patients; B, poorly differentiated EC patients)

	**OS**			**DFS**			**PFS**		
**Variable**	**HR**	**95% CI**	***P*-value**	**HR**	**95% CI**	***P*-value**	**HR**	**95% CI**	***P-value***
*(A)*									
*Lymph node involvement*									
Positive *vs* negative	0.67	0.07–6.11	0.72	1.20	0.14–10.26	0.87	0.53	0.06–5.06	0.58
									
*Histological type*									
Non-endometrioid *vs* endometrioid	4.89	1.09–21.81	0.01	2.37	0.64–8.75	0.22	3.30	0.80–13.56	0.04
									
*FIGO stage*									
III+IV *vs* I+II	20.32	1.79–230.40	0.04	3.84	0.44–33.47	0.20	11.72	1.13–121.31	0.09
									
*HE4 serum levels (tertiles)*									
High *vs* low	2.84	0.54–15.01	0.22	2.80	0.65–12.15	0.17	2.76	0.47–16.14	0.26
Medium *vs* low	5.35	0.80–35.67	0.08	3.05	0.69–13.51	0.14	3.18	0.53–19.17	0.21
									
*(B)*									
*Lymph node involvement*									
Positive *vs* negative	0.55	0.05–5.58	0.62	1.32	0.14–12.30	0.81	0.54	0.05–6.08	0.62
									
									
*Histological type*									
Non-endometrioid *vs* endometrioid	1.06	0.21–5.29	0.04	0.75	0.16–3.55	0.31	0.98	0.22–4.39	0.07
									
*FIGO stage*									
III+IV *vs* I+II	14.38	1.13–183.28	0.94	3.36	0.32–35.47	0.71	10.06	0.81–125.71	0.98
									
*HE4 serum levels (tertiles)*									
High *vs* low	6.85	1.02–46.17	0.04	17.00	1.89–153.24	0.01	9.81	1.01–95.26	0.04
Medium *vs* low	4.98	0.80–31.06	0.08	5.10	0.50–51.00	0.17	6.74	0.72–62.54	0.09

Abbreviations: CI=confidence interval; DFS=disease-free survival; EC=endometrial carcinoma; FIGO=International Federation of Gynecology and Obstetrics; HE4=human epididymis protein 4; HR=hazards ratio; OS=overall survival; PFS=progression-free survival; WHO=World Health Organisation.
